# Research trends in cardiac rehabilitation following COVID-19: A cross-sectional bibliometric study

**DOI:** 10.1097/MD.0000000000046028

**Published:** 2025-11-21

**Authors:** Siddig Ibrahim Abdelwahab, Manal Mohamed Elhassan Taha, Hadeel R. Bakhsh, Monira I. Aldhahi

**Affiliations:** a Health Research Centre, Jazan University, Jazan, Saudi Arabia; b Department of Rehabilitation Sciences, College of Health and Rehabilitation Sciences, Princess Nourah bint Abdulrahman University, Riyadh, Saudi Arabia.

**Keywords:** cardiac rehabilitation, dynamicity, scienctemtrics, scopus, thematically-based analysis

## Abstract

This bibliometric analysis assesses scientific progress, spatial distribution, keyword trends, thematic evolution, and research gaps in cardiac rehabilitation research (CRR), with a focused appraisal of the post-COVID-19 era (2020–2023). Scopus-indexed publications from 1948 to 2023 and 2020 to 2023 were analyzed using VOSviewer (v1.6.19) and Biblioshiny (v2.0.2). The study was strengthening the reporting of observational studies in epidemiology-compliant. A total of 9173 CRR documents were identified, showing sustained exponential growth over time. The Journal of Cardiopulmonary Rehabilitation and Prevention emerged as the leading source. The United State, Canada, and the United Kingdom led global output and collaboration networks. Core keywords included “cardiac rehabilitation,” “coronary artery disease,” “myocardial infarction,” “exercise,” and “rehabilitation.” Post-COVID-19 analyses revealed a discernible thematic shift, with emerging clusters and hot themes centered on “age,” “primary care,” “heart transplant,” and “exercise”. This first comprehensive bibliometric overview of CRR maps long-term growth, geographic leaders, evolving themes, and research gaps, and highlights a reorientation of priorities in the post-COVID-19 era to inform future research directions.

## 1. Introduction

Cardiac rehabilitation is a multifaceted program that aims to aid individuals in recovering from cardiovascular events and enhancing their overall cardiovascular health. It encompasses a range of interventions including medical supervision, exercise training, education, and counseling, all designed to mitigate the risk of future cardiac complications and foster well-being. The efficacy of cardiac rehabilitation has been widely recognized and numerous studies have demonstrated its positive impact on patient outcomes and quality of life.^[1,2]^ By employing a holistic approach, this program supports individuals in their journey toward recovery and empowers them to actively engage in managing their cardiovascular health.^[2,3]^

Medical evaluation is the initial step in cardiac rehabilitation, enabling healthcare professionals to assess a patient’s cardiac condition, identify risk factors, and customize an appropriate exercise and treatment plan.^[4]^ The exercise training component, a cornerstone of cardiac rehabilitation, encompasses aerobic exercises such as walking, cycling, swimming, and strength training. These exercises are carefully tailored to individual capabilities and are closely supervised by healthcare providers to ensure safety and effectiveness.^[5–7]^

Education and counseling are pivotal for empowering patients to understand their cardiac conditions, manage risk factors, and improve self-care practices. Patients receive comprehensive education on heart health, including guidance on lifestyle modifications, medication management, and recognition of warning signs for future cardiac events.^[5]^ Psychological support is provided to address the emotional impact of cardiac events, manage stress, and address any anxiety or depression that may arise.^[8]^

Nutritional guidance is another vital aspect of cardiac rehabilitation, because a healthy diet significantly contributes to cardiovascular well-being patients receive personalized recommendations for adopting a balanced diet, reducing sodium and saturated fat intake, and effectively managing weight.^[9]^ Additionally, medication management ensures that patients understand the purpose and proper administration of prescribed medications, promote adherence, and minimize potential side effects.^[10]^ Cardiac rehabilitation programs can help patients modify their lifestyles and mitigate future cardiovascular risks.^[11,12]^ Lifestyle changes, including increased physical activity, smoking cessation, and dietary improvements have been emphasized to foster long-term cardiovascular health.

Overall, cardiac rehabilitation is a comprehensive and evidence-based program that encompasses medical evaluation, exercise training, education, counseling, and risk factor modification. With its multidimensional approach, cardiac rehabilitation enables patients to achieve improved cardiovascular fitness, symptom management, and enhanced overall well-being. Despite the growing volume of literature, a research gap exists in understanding the global scientific landscape and evolving priorities in cardiac rehabilitation, particularly in the context of the post-COVID-19 era. The main objective of this study is to conduct a comprehensive bibliometric analysis of cardiac rehabilitation research (CRR) from 1948 to 2023, using data extracted from the Scopus database. Bibliometric analysis was selected as the knowledge synthesis method because it allows for a systematic and quantitative evaluation of large bodies of literature, enabling the identification of influential contributors, research trends, and thematic developments in the field. A timely bibliometric analysis can guide funding allocation, workforce planning, and program redesign after pandemic-driven disruptions to cardiac rehabilitation, including tele-rehabilitation expansion and changing patient needs. By mapping outputs, themes, and gaps, it benchmarks global activity to inform priorities. Prior bibliometric studies were narrow or dated; we broaden coverage, incorporate post-COVID-19 dynamics, and apply complementary analytic tools and metrics. The study aims to evaluate publication trends, identify the most prolific authors, institutions, and countries, and examine keyword co-occurrence and thematic clusters using scientometric tools such as VOSviewer and Biblioshiny. Additionally, it seeks to explore the thematic evolution of CRR in the post-COVID-19 era (2020–2023) compared to earlier periods, identify emerging research priorities, and uncover knowledge gaps. The findings are intended to provide valuable insights for researchers, clinicians, and policymakers to guide future investigations, improve cardiac rehabilitation practices, and inform evidence-based policy and program development.

## 2. Materials and methods

### 2.1. Database selection, terms generations, and inclusion criteria

**Figure 1.**
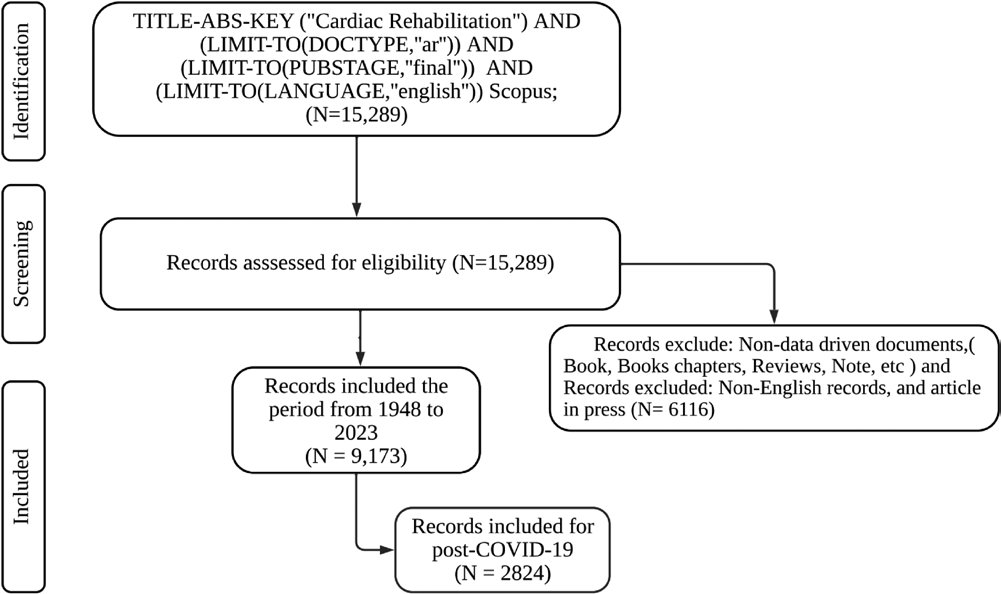
Search strategy based on PRISMA guidelines. Data on post-COVID-19 were extracted in January 2020. COVID-19 = coronavirus disease 2019.

The search strategy aimed to comprehensively retrieve pertinent articles related to cardiac rehabilitation (CR) using both medical subject headings and the Scopus database. Scopus was selected for its comprehensive multidisciplinary coverage, inclusion of high-impact journals, robust citation tracking, and advanced analytical features, ensuring consistent metadata quality and reliable bibliometric mapping, which make it well-suited for capturing global CRR trends.^[13]^ In the medical subject headings database, the terms “cardiac rehabilitation” and “heart rehabilitation” were applied to identify articles specifically indexed under these headings. In Scopus, the following search string was used verbatim: (TITLE-ABS-KEY (“Cardiac Rehabilitation”) AND (LIMIT-TO (DOCTYPE, “ar”)) AND (LIMIT-TO (PUBSTAGE, “final”)) AND (LIMIT-TO (LANGUAGE, “English”)). This search string was executed on October 25, 2023, ensuring inclusion of all records published from database inception through 2023. For the post-COVID-19 subset, data were extracted for the period January 1, 2020 to December 31, 2023. To ensure transparency and reproducibility, the search was restricted to peer-reviewed journal articles in English, excluding conference abstracts, gray literature, editorials, letters, and notes. All records retrieved were subjected to screening and eligibility assessment following PRISMA guidelines,^[14]^ with a detailed PRISMA flow diagram (Fig. 1) documenting the identification, screening, eligibility, and inclusion processes. Three time-slice divisions – 1948 to 2010, 2011 to 2019, and 2020 to 2023 – were used to reflect historical publication trends, pre-COVID-19 developments, and the post-COVID-19 period, respectively. This bibliometric study did not require ethical approval, as it analyzed publicly available secondary data.

### 2.2. Quality control

Authors SIA and MMET assessed the significance of the most frequently referenced publications and their correlation with the CRR. A preliminary analysis was performed prior to the final analysis to ensure attainment of the study objectives.

### 2.3. Data extraction and analysis

The comma-separated values and BibTeX files were extracted from the Scopus database and analyzed using the VOSviewer and Biblioemtrix software.^[15,16]^ VOSviewer, a widely used software tool for visualizing and exploring bibliometric networks, was employed to analyze and visualize the relationships between different entities, such as authors, keywords, and publications, based on the bibliographic data extracted. Researchers were able to identify patterns, clusters, and trends within the data, thereby gaining insight into research areas, collaborations, and emerging topics in the field of cardiac rehabilitation. VOSviewer was used with fractionalization normalization and the LinLog/modularity clustering algorithm. Thresholds for co-authorship, co-occurrence, citation, and bibliographic coupling were applied as per dataset size. Similarly, Biblioemtrix, a bibliometric analysis software, was used to assess the scholarly impact of publications and authors. An evaluation of the productivity, citation impact, and collaboration patterns of individual researchers or research groups based on the extracted bibliographic data was conducted. Biblioshiny parameters included minimum keyword frequency, network layout optimization, and thematic map centrality/density settings.

In particular, thematic map analysis was used to explore the conceptual structure of the field. This involved calculating Callon centrality – a metric that reflects the degree of interaction between a theme and other themes in the field (i.e., its importance) – and Callon density, which measures the internal strength and development of a theme (i.e., how cohesive and mature it is). Themes with high centrality and high density are classified as motor themes, indicating well-developed and highly relevant research areas. By leveraging the capabilities of VOSviewer and Bibliometrix, we conducted in-depth analyses of the Scopus data, uncovering valuable insights into the structure of the research field, influential authors, key research topics, and the evolution of themes in CRR.

## 3. Bradford’s Law analysis

To identify the core journals in CRR, Bradford’s Law was applied using the bibliometric data extracted from the Scopus database.^[17]^ The analysis was conducted using the Bibliometrix R-package, which organizes the sources by productivity (i.e., the number of articles published). Journals were ranked in descending order based on their output, and then grouped into 3 Bradford zones: zone 1 (core journals), zone 2, and zone 3, each contributing approximately the same number of articles. This method allowed us to visualize the skewed distribution of scholarly productivity and identify the most influential journals in the field.

## 4. Lotka’s Law analysis

Lotka’s Law was applied to assess author productivity within the CRR domain. Using the same dataset from Scopus, author-level data were processed using Bibliometrix, which generated frequency distributions showing how many authors published a given number of documents. The percentage of authors contributing 1, 2, 3, or more publications was calculated and compared against Lotka’s theoretical distribution, where the number of authors publishing n papers is approximately 1/n² of those publishing a single paper.^[18]^ Quantitative reports and data visualizations were generated to analyze publication patterns, citation networks, and research impacts on the CRR. By leveraging the capabilities of VOSviewer and Biblioemtrix, we conducted in-depth analyses of the extracted Scopus data, uncovering valuable insights into the structure of the research field, influential authors, key research topics, and impact of CRR publications.

## 5. Results

### 5.1. *Annual growth (1948–2023*)

**Figure 2.**
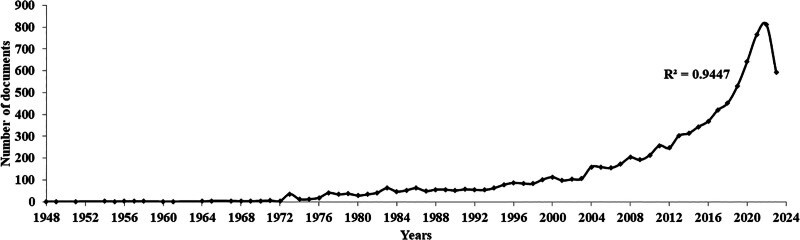
Annual growth of research in cardiac rehabilitation. The growth curve was observed to fit exponential regression with an *R*^2^ of 0.9447.

Figure 2 shows the annual production in the CRR based on a dataset of 9173 documents spanning the period 1948 to 2023. From 2000 onwards, publications represented approximately 83% of the total dataset, indicating a significant focus on CRR in recent years. Furthermore, in the last decade (2014–2023), publications accounted for 57.17% of the total dataset, reflecting a substantial proportion of research activity in this field. The growth curve fit an exponential regression with an *R*^2^ value of 0.9447. Exponential regression suggests that the growth of publications in this field has been rapid and accelerating, highlighting the increasing interest and focus on CRR.

### 5.2. Contributing scholars

In total, 32,630 authors were included in the dataset. Among these, 591 authors contributed to single-authored documents. This indicates that a small portion of the authors in the dataset authored documents without coauthors. Several authors have emerged as the most prolific contributors in the field of CRR. Grace stands out with 190 documents affiliated with York University in Canada. Oh closely follows 100 documents and is affiliated with the University of Toronto, Canada. Doherty, P., affiliated with the University of York in the United Kingdom, has contributed 77 documents, while Ades, P.A., from the University of Vermont in the United State, has 69 publications to their credit. Lavie, C.J., affiliated with the Ochsner Heart and Vascular Institute in the United State, made significant contributions with 66 documents. The notable affiliations and extensive publication records of these authors highlight their influential roles in advancing the field of CRR. Table 1 lists the most locally impactful researchers in the CRR field. These metrics provide insights into authors’ research impact, including citation counts, publication numbers, and the starting years of their publication records. Lavie, C.J. stands out with an Hirsch index (*H*-index) of 41, Gini index (*G*-index) of 66, and median index of 1.242. With 4996 citations and 66 publications since 1991, Lavie has had a significant impact. Milani, R.V. follows closely with an *H*-index of 39, a *G*-index of 58, and an median index of 1.182. Ades, P.A., with an *H*-index of 36 and a *G*-index of 68, has garnered 5864 citations from 68 publications since 1986. Grace, S.L., with an *H*-index of 36 and a *G*-index of 56, has an impressive publication record of 191 papers since 2002, resulting in 4494 citations. Other notable authors include Thomas, R.J. (*H*-index: 29), Taylor, R.S. (*H*-index: 28), and Oh, P. (*H*-index: 26). The contributions of these authors have played a vital role in advancing the CRR, as evidenced by their impactful metrics.

**Table 1:** Most local impactful scholars in CRR*.

Authors	*H*_index	*G*_index	*M*_index	TC	NP	PY_start
Lavie, C.J.	41	66	1.242	4996	66	1991
Milani, R.V.	39	58	1.182	4742	58	1991
Ades, P.A.	36	68	0.947	5864	68	1986
Grace, S.L.	36	56	1.636	4494	191	2002
Thomas, R.J.	29	59	0.806	4107	59	1988
Taylor, R.S.	28	67	1.400	5214	67	2004
Oh, P.	26	42	1.625	2110	100	2008
Savage, P.D.	25	38	1.000	1504	45	1999
Squires, R.W.	25	45	0.595	2055	47	1982
Dendale, P.	23	51	1.211	2820	51	2005

CRR = cardiac rehabilitation research, *H*-index = Hirsch index, *G*-index = Gini index, *M*-index = median index.

* The *H*-index represents the number of papers with at least h citations, the *G*-index measures the cumulative impact of an author’s publications, and the *M*-index is a measure of collaboration. The TC refers to the total number of citations received by an author, NP is the number of publications, and PY_start indicates the year when the author started publishing in the field.

#### 5.2.1. Role of institutions and countries

University of Toronto was the most prominent and contributed significantly to the field. The University Health Network, connected to the University of Toronto, closely monitored 238 events and underlined their importance. The University of Toronto’s Toronto Rehabilitation Institute was also present 194 times. York University was also significant, with 191 documents. Thus, the Mayo Clinic, known for its research, appears 140 times in the dataset. These data demonstrate the active involvement and influence of these institutions in this research, indicating their vital contributions.

**Figure 3.**
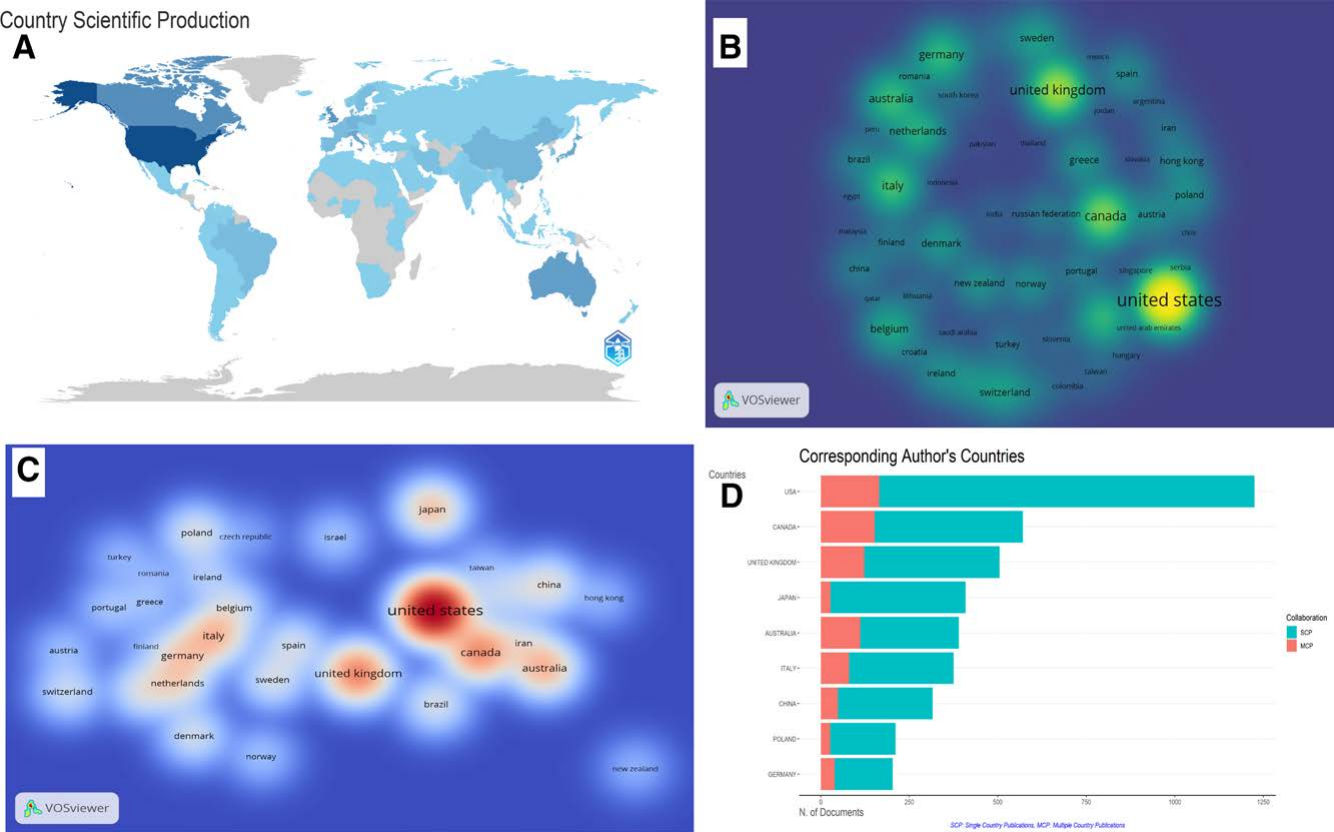
Role of countries in CRR. (A) The top prolific countries in CRR. (B) The most cited countries in CRR as mapped using VOSviewer software. (C) Depicts the collaboration in CRR, showcasing the mapping of collaborative networks using VOSviewer software. (D) Introduces the concept of single- (SCP) and multiple-country publications (MCP), with the MCP ratio serving as a measure of collaboration. CRR = cardiac rehabilitation research, MCP = multiple country publications, SCP = single country publications.

Figure 3A provides an insight into the top prolific countries in the CRR. The United States tops the list, with 6339 publications. Canada ranked second with 2983 publications, followed by the United Kingdom with 2541 publications. Australia and Japan also made significant contributions with 2194 and 1884 publications, respectively. Italy, China, Germany, the Netherlands, and Poland were the top 10 countries. Notably, Western countries dominate the list, with the exceptions of China and Japan.

Figure 3B highlights the countries most cited in CRR. The United State leads with a total of 32,463 citations, followed by the United Kingdom (16,176 citations) and Canada (13,912 citations). Italy and Australia also received significant citation counts, with 8276 and 7466 citations, respectively. The top 5 countries contributed to 61.21% of the total citations.

Figure 3C depicts the collaboration in CRR, showing the mapping of collaborative networks using the VOSviewer software. The United States, United Kingdom, Italy, and Germany are among the top collaborating countries. Specifically, the United State has established 355 research links in various countries. Notably, the United State collaborated with Canada on 187 occasions, 86 with the United Kingdom, and 82 with Italy. Figure 3D introduces the concepts of single and multiple-country publications (MCP), with the MCP ratio serving as a measure of collaboration. Brazil has the highest MCP ratio (0.292) among the 10 most collaborative countries, indicating substantial involvement in international research collaborations. These findings shed light on the research productivity, citation impact, and collaborative efforts of various countries in cardiac rehabilitation, providing valuable insights for understanding the global landscape of this domain.

## 6. Bradford’s Law

**Figure 4.**
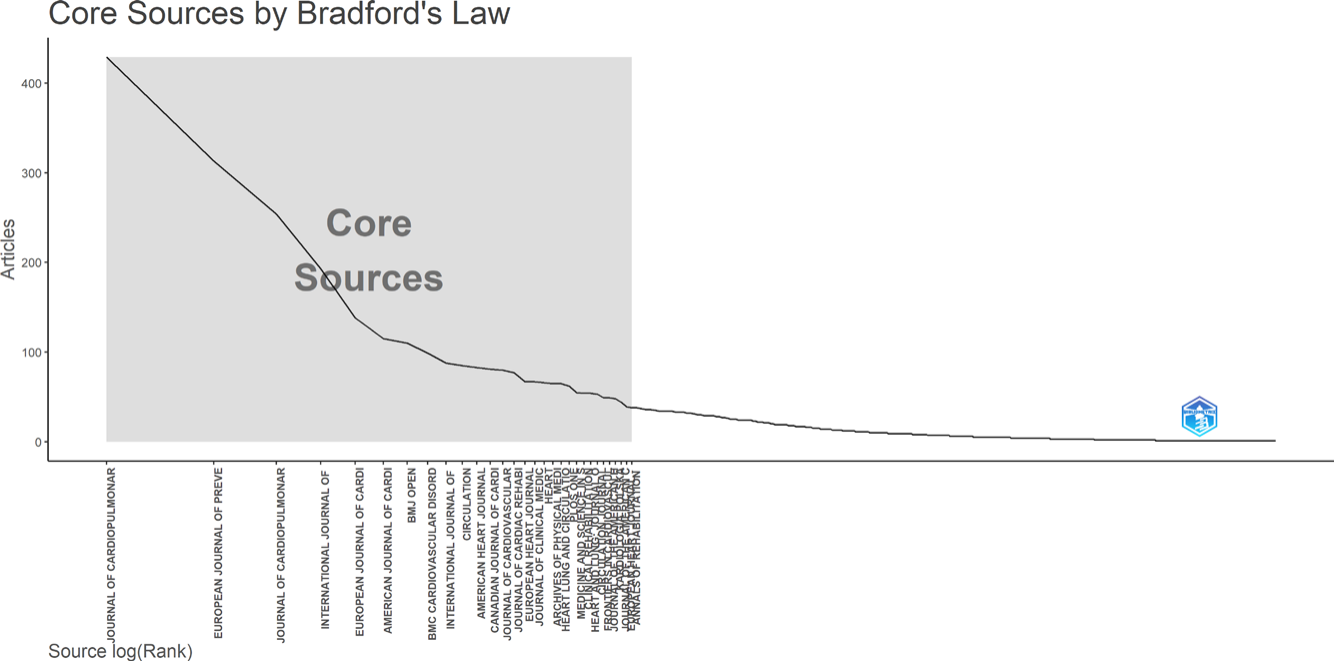
Bradford’s Law. Core journals in cardiac rehabilitation research identified using Bradford’s Law. The plot categorizes 2140 journals into 3 productivity zones. zone 1 includes 30 core journals contributing the majority of articles, followed by zone 2 (221 journals) and zone 3 (1689 journals), illustrating the uneven distribution of research output across publication sources in this field. This figure was generated using the Bibliometrix application and the BibTex data file.

As illustrated in Figure 4, the application of Bradford’s Law provides insight into the dispersion of scholarly articles across various journals in the field of CRR. Bradford’s Law is a bibliometric principle that states that if journals are arranged in order of decreasing productivity on a specific topic, they can be divided into a core set of journals most frequently publishing on the subject (zone 1), followed by a second zone with the same number of articles spread across more journals, and a third zone with an even larger number of journals producing the same article volume. In our study, 2140 journals contributed to the field of CRR, but only a small number of journals dominated the publication landscape. Specifically, zone 1 consisted of only 30 core journals, which accounted for 3020 articles and included leading sources, such as the Journal of Cardiopulmonary Rehabilitation and Prevention, European Journal of Preventive Cardiology, and International Journal of Cardiology. These journals serve as foundational sources for disseminating CRR-related research and are central to knowledge exchange in the field. In comparison, zone 2 comprised 221 journals, and zone 3 encompassed a significantly larger pool of 1689 journals, each contributing fewer publications. This skewed distribution reflects the typical Bradford pattern and highlights the importance of targeting core journals for visibility and impact in CRR scholarships. These findings are useful for guiding researchers in selecting high-impact journals, librarians in making informed subscription decisions, and policymakers in understanding knowledge dissemination patterns in cardiac rehabilitation.

### 6.1. Source dynamics

**Figure 5.**
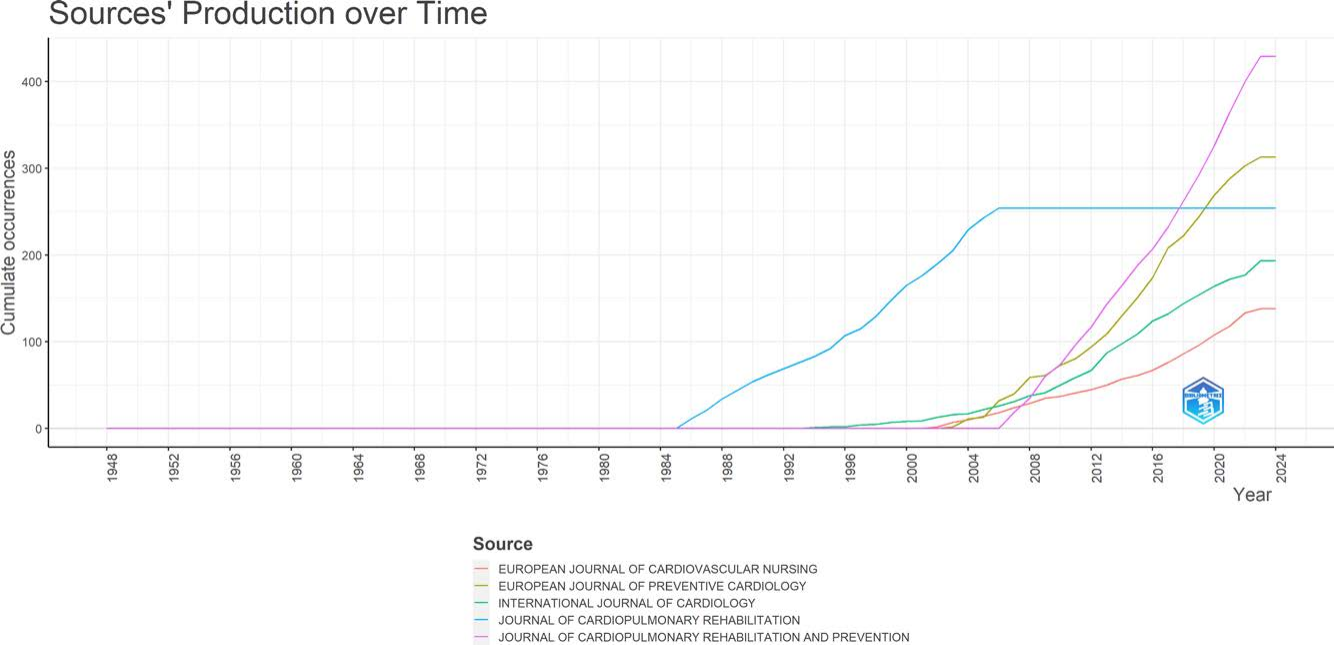
The dynamics of journals in CRR can be observed through the publication counts. This figure illustrates the cumulative publication trends over time for the top 5 journals contributing to cardiac rehabilitation research. The Journal of Cardiopulmonary Rehabilitation and Prevention shows the highest growth, especially post-2010. Other notable contributors include the European Journal of Preventive Cardiology and the International Journal of Cardiology, which have exhibited steady increases, indicating rising interest and scholarly output in this domain. This figure was generated using the Bibliometrix application and the BibTex data file. CRR = cardiac rehabilitation research.

Among the 1941 journals, the top accommodating journals for CRR included the Journal of Cardiopulmonary Rehabilitation and Prevention, which accommodated 429 articles, the European Journal of Preventive Cardiology with 313 articles, and the Journal of Cardiopulmonary Rehabilitation with 254 articles. Circulation, a highly respected journal in cardiovascular research, leads the way with an impressive total of 17,114 citations. The European Journal of Preventive Cardiology and Circulation has emerged as one of the most cited journals. It has received a notable total of 12,979 citations, highlighting its significance in the field. The dynamics of journals in the CRR can be observed through publication counts for specific years (Fig. 5).

Among the journals mentioned, the Journal of Cardiopulmonary Rehabilitation and Prevention shows a consistent increase in publications over the years, reaching 429 articles by 2023. The European Journal of Preventive Cardiology also demonstrated a steady rise in publications, reaching 313 articles in 2023. The Journal of Cardiopulmonary Rehabilitation remained consistent, with 254 publications throughout the study period. The International Journal of Cardiology has shown a gradual increase, reaching 193 publications in 2023. The European Journal of Cardiovascular Nursing exhibited a similar pattern, with 138 publications by 2023. These dynamics reflect the continued research activity and interest in cardiac rehabilitation, as evidenced by the publication output in these journals.

### 6.2. Three-field plot

**Figure 6.**
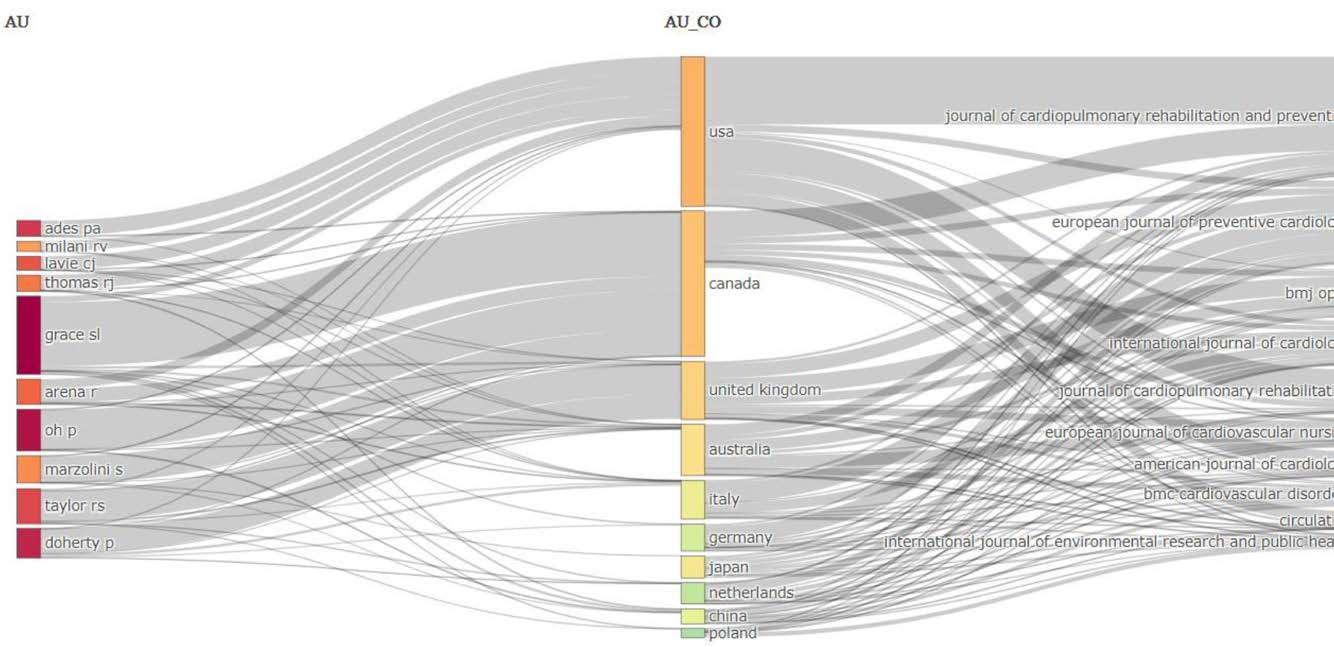
Sankey diagram illustrating the 3-field plot connecting top contributing authors (AU), their affiliated countries (AU_CO), and the journals (SO) where their cardiac rehabilitation research was published. Authors like Grace SL and Ades PA contributed significantly, with the United State and Canada being the most productive countries. The Journal of Cardiopulmonary Rehabilitation and Prevention received the highest volume of publications. This figure was generated using the Bibliometrix application and the BibTex data file.

A 3-field plot, or Sankey diagram, is a visual representation that illustrates the flow or distribution of data among the 3 categories. It consists of 3 columns or fields with arrows or lines connecting the categories to represent their relationships or interactions.^[19]^ In the context of CRR, the plot displays the connections among the top 10 authors, countries, and journals regarding contributions, collaborations, and publishing trends. This visual representation provides insight into the distribution and interactions within the field, offering a clear overview of the relationships and dynamics among these 3 categories. Based on the data in Figure 6, Grace preserves the first spot in publishing in the top-10 journals. The United State and Canada have published their CRR in all journals.

### 6.3. The Lotka’s Law

**Figure 7.**
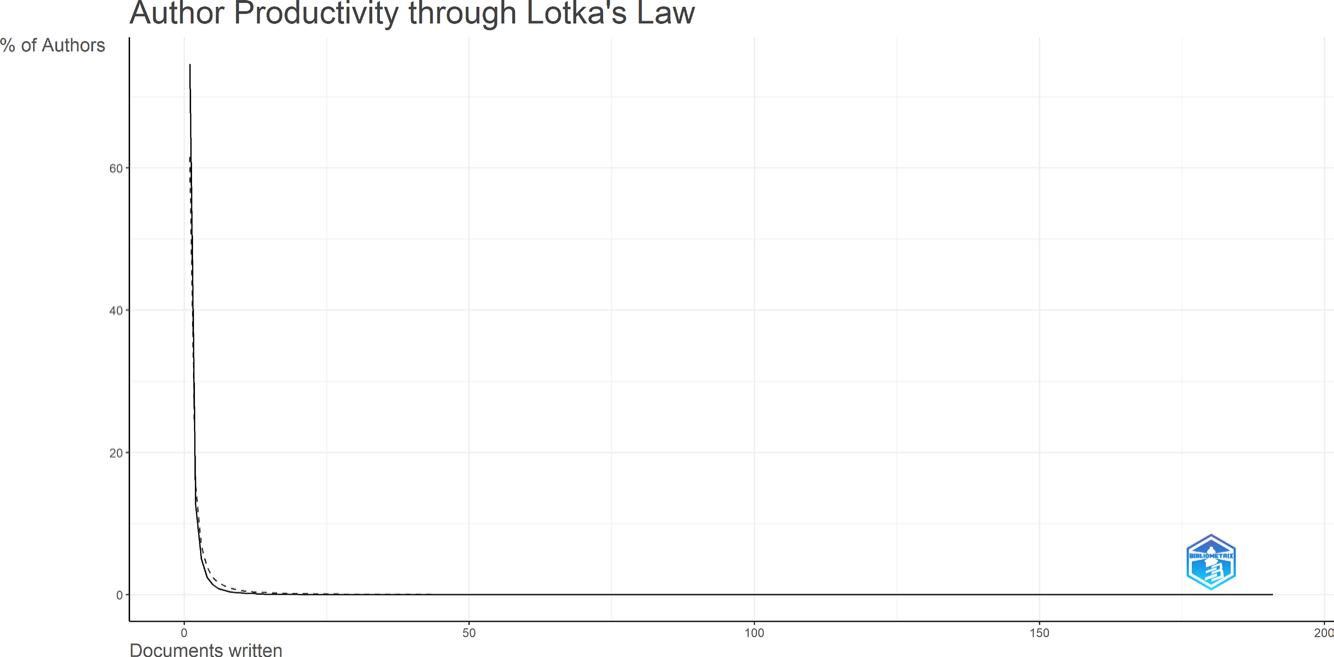
Author productivity through Lotka’s Law. The graph illustrates the distribution of authors based on the number of documents published in cardiac rehabilitation research. Consistent with Lotka’s Law, the majority of authors contributed only one publication, while a small percentage produced multiple papers, highlighting the significant impact of a limited number of highly productive researchers in the field. This figure was generated using the Bibliometrix application and the BibTex data file.

The Lotka’s Law distribution shown in Figure 7 demonstrates the productivity pattern among authors in CRR. According to the data, approximately 74.6% of authors published only one document, while 12.6% published 2, and progressively fewer authors published 3 or more. This distribution reflects the classic inverse square law, in which a small proportion of authors contribute a large share of publications. This trend aligns with Lotka’s Law, which postulates that the number of authors publishing n papers is approximately 1/n² of those publishing 1 paper. These results emphasize the dominance of a few prolific contributors in shaping the field, whereas the majority of researchers contribute on a more limited basis. This analysis underscores the importance of recognizing both highly productive researchers and the broader scientific community supporting CRR.

### 6.4. Conceptual structure: keywords co-occurrence

**Figure 8.**
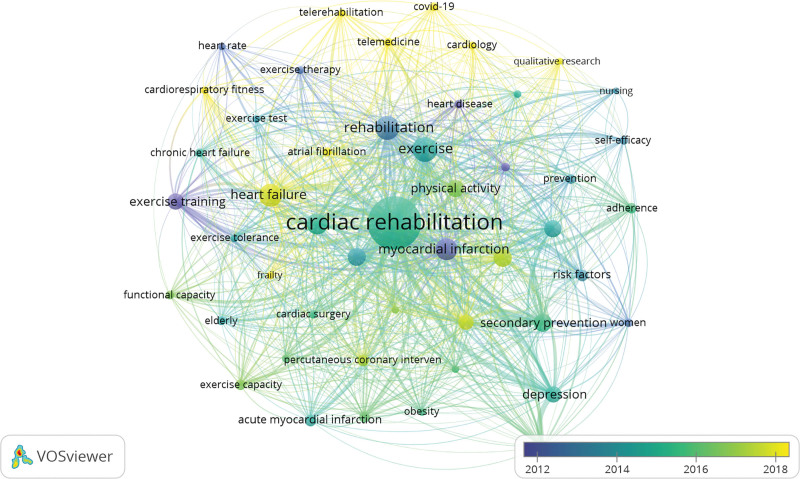
Mapping of conceptual structure: keywords co-occurrence. The bar shows the emergence of keywords according to their time of appearance in the CRR. Yellow nodes are the most recent keywords in CRR. Nodes represent the frequency of the keywords. The mapping of keywords was conducted using VOSviewer software. CRR = cardiac rehabilitation research.

The conceptual structure of keyword co-occurrence in CRR can be observed through the most frequent keywords and their respective occurrences and percentages (Table 2 and Fig. 8). The table shows the top keywords and their frequencies in the field. There were 8893 instances of author keywords chosen by the authors to represent the main concepts of their research. The most frequent keyword is “cardiac rehabilitation,” with 2944 occurrences, followed by “coronary artery disease” (791 occurrences) and “myocardial infarction” (664 occurrences). Other significant keywords include “exercise,” “rehabilitation,” “heart failure,” “cardiovascular disease,” “exercise training,” “secondary prevention,” and “quality of life,” among others. These keywords reflect the key areas of focus and research interest within the CRR, covering aspects such as disease conditions, interventions, outcomes, and associated factors. Analyzing keyword co-occurrence can provide insights into the relationships and patterns of research topics in cardiac rehabilitation.

**Table 2:** Most frequent keywords in CRR.

1948–2023			2020-2023		
Keywords	Occurrences	Percentage	Keywords	Occurrences	Percentage
Cardiac rehabilitation	2944	9.43	Cardiac rehabilitation	1081	8.77
Coronary artery disease	791	2.53	Heart failure	262	2.13
Myocardial infarction	664	2.13	Exercise	244	1.98
Exercise	635	2.04	Myocardial infarction	198	1.61
Rehabilitation	608	1.95	Cardiovascular disease	214	1.74
Heart failure	532	1.70	Coronary artery disease	148	1.20
Cardiovascular disease	498	1.60	Rehabilitation	131	1.06
Exercise training	404	1.29	Physical activity	126	1.02
Secondary prevention	355	1.14	Secondary prevention	123	1.00
Quality of life	333	1.07	Coronary heart disease	115	0.93
Physical activity	308	0.99	Acute coronary syndrome	106	0.86
Depression	280	0.90	Quality of life	104	0.84
Acute coronary syndrome	239	0.77	Depression	88	0.71
Anxiety	164	0.53	COVID-19	79	0.64
Risk factors	137	0.44	Exercise training	122	0.99
Percutaneous coronary intervention	132	0.42	Anxiety	63	0.51
Mortality	118	0.38	Percutaneous coronary intervention	57	0.46
Adherence	111	0.36	Adherence	47	0.38
Prevention	91	0.29	Cardiorespiratory fitness	45	0.36
Chronic heart failure	90	0.29	Case report	43	0.35
Cardiac surgery	89	0.29	Rehabilitation medicine	43	0.35
Elderly	88	0.28	Mortality	42	0.34

CRR = cardiac rehabilitation research.

### 6.5. Thematic map

**Figure 9.**
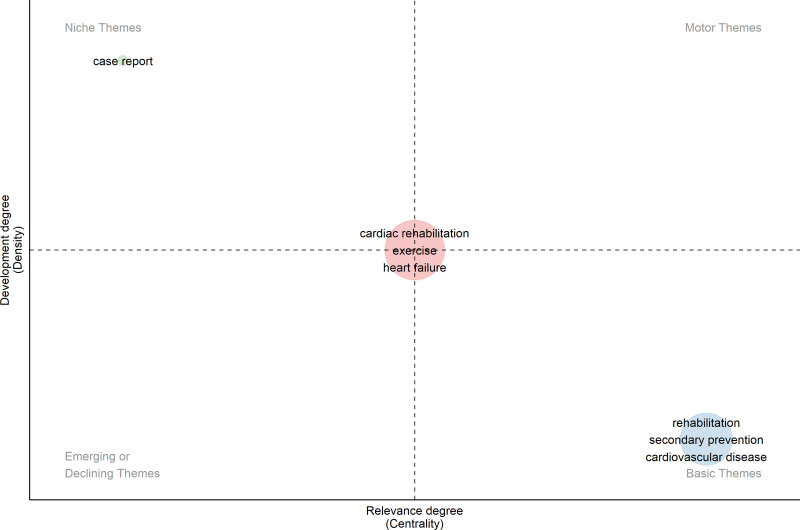
Thematic map of cardiac rehabilitation research, categorizing themes by Callon centrality (relevance) and density (development). Core themes such as “cardiac rehabilitation,” “exercise,” and “heart failure” appear centrally positioned, indicating strong development and relevance. “Rehabilitation” and “cardiovascular disease” are basic themes, while “case report” appears as a niche theme. No emerging or declining themes were identified in this map.

Figure 9 presents a thematic map that categorizes research clusters in cardiac rehabilitation. Table 3 provides additional information on these clusters, including callon centrality, callon density, keywords associated with each theme, and classification. In the thematic map, the *x*-axis (Callon centrality) reflects a theme’s importance – its degree of connection with other themes (sum of external link strengths). The *y*-axis (Callon density) reflects a theme’s maturity – its internal cohesion (intra-cluster link strength). Quadrants: motor (high–high), basic/transversal (high–low), niche (low–high), emerging/declining (low–low). In Figure 9, a thematic map of the CRR is constructed using the keywords of the author. This indicates that the clustering and classification of themes within the map were based on keywords chosen by the authors to represent the main concepts or topics of CRR. The thematic map and associated table help identify and categorize the different clusters of research within the CRR based on their centrality, density, and keywords. This classification provides insights into the main themes and areas of focus of the CRR.

**Table 3:** Clusters, centrality, density, and keywords, and classification.

Clusters	Callon centrality	Callon density	Keywords of the theme	Classification
Cardiac rehabilitation	0.05	1.71	Cardiac rehabilitation, exercise, heart failure, myocardial infarction, coronary artery disease, quality of life, exercise training, acute coronary syndrome, acute myocardial infarction, percutaneous coronary intervention, exercise capacity, mortality, chronic heart failure, cardiac surgery, elderly, COVID-19, functional capacity, atrial fibrillation, obesity, prognosis, cardiorespiratory fitness, health-related quality of life, ischemic heart disease, exercise tolerance, heart rate, exercise test, exercise therapy, frailty, diabetes, hypertension, exercise testing, ischemic heart disease, blood pressure, gender, echocardiography, cardiopulmonary exercise testing, heart rate variability, stroke, coronary artery bypass grafting, meta-analysis, outcomes, resistance training	Centered
Rehabilitation	0.08	1.50	Rehabilitation, secondary prevention, cardiovascular disease, coronary heart disease, physical activity, depression, anxiety, cardiovascular diseases, risk factors, adherence, prevention, heart disease, women, telemedicine, coronary disease, self-efficacy, telerehabilitation, patient education, cardiology, nursing, qualitative research, randomized controlled trial, rehabilitation medicine, cardiac, return to work, self-management, barriers	Basic
Case report	0.00	2.00	Case report	Niche

COVID-19 = coronavirus disease 2019.

## 7. Cardiac rehabilitation

This cluster has a Callon centrality of 0.05 and a Callon density of 1.71. The keywords associated with this theme included cardiac rehabilitation, exercise, heart failure, myocardial infarction, coronary artery disease, quality of life, and exercise training. In the thematic map, this theme is positioned at the center, surrounded by the “basic,” “motor,” “niche,” and “emerging” themes. This central placement suggests that cardiac rehabilitation is a focal point within the thematic landscape, exerting influence and connecting with other themes. The proximity to the “basic” theme indicates its fundamental role, while its position between the “motor” and “niche” themes suggests its involvement in both dynamic aspects and specialized areas. Furthermore, being adjacent to the “emerging” theme suggests that cardiac rehabilitation is actively evolving and exploring new developments within the field. Overall, this central positioning emphasizes the significance and multidimensional nature of cardiac rehabilitation within the broader context of the thematic map.

## 8. Rehabilitation

This cluster has a Callon centrality of 0.08 and a Callon density of 1.50. Keywords associated with this theme included rehabilitation, secondary prevention, cardiovascular disease, coronary heart disease, physical activity, depression, and anxiety. This cluster was classified as “basic,” suggesting a fundamental role in CRR.

## 9. Case report

This cluster has a Callon centrality of 0.00 and a Callon density of 2.00. The theme is “Case Report.” This cluster is classified as “niche,” indicating its specialized nature within CRR, focusing specifically on case reports.

### 9.1. Dynamicity of CRR

**Figure 10.**
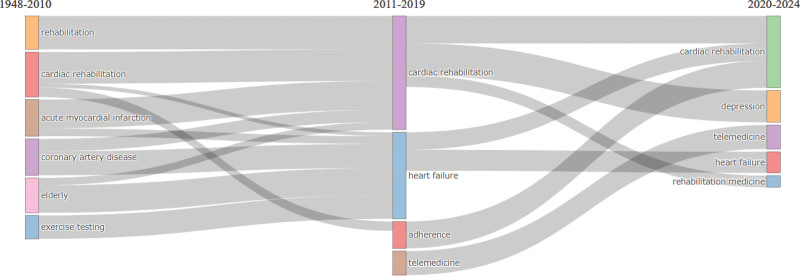
Dynamicity of cardiac rehabilitation research (CRR) from 1948 to 2024. The figure illustrates the evolution of key research themes across 3 time periods. Early themes like “acute myocardial infarction” and “exercise testing” gradually gave way to contemporary focuses such as “heart failure,” “depression,” “telemedicine,” and “rehabilitation medicine,” reflecting a shift toward holistic, tech-integrated, and mental health-aware CRR. CRR = cardiac rehabilitation research.

Figure 10 shows the thematic evolution of CRR over different periods. This figure shows the transitions of themes from 1 period to another. From the period of 1948 to 2010, the dominant themes were “acute myocardial infarction,” “cardiac rehabilitation,” “coronary artery disease,” “elderly,” “exercise testing,” and “rehabilitation.” In the period of 2011 to 2019, the themes shifted to “cardiac rehabilitation,” “heart failure,” “adherence,” “depression,” “rehabilitation medicine,” and “telemedicine.” Moving forward to the period of 2020 to 2024, the themes that persisted and emerged were “cardiac rehabilitation,” “depression,” “rehabilitation medicine,” “telemedicine,” and “heart failure.” This thematic evolution suggests a transition over time in focus and research interest within the field of cardiac rehabilitation. The earlier emphasis on acute myocardial infarction, coronary artery disease, and exercise testing shifted towards a broader exploration of cardiac rehabilitation, heart failure, adherence, and the incorporation of telemedicine from 2011 to 2019 period. In the more recent period of 2020 to 2024, the themes of depression and rehabilitation medicine gained prominence, along with continued focus on cardiac rehabilitation, heart failure, and telemedicine.

### 9.2. Emerging CRR

**Figure 11.**
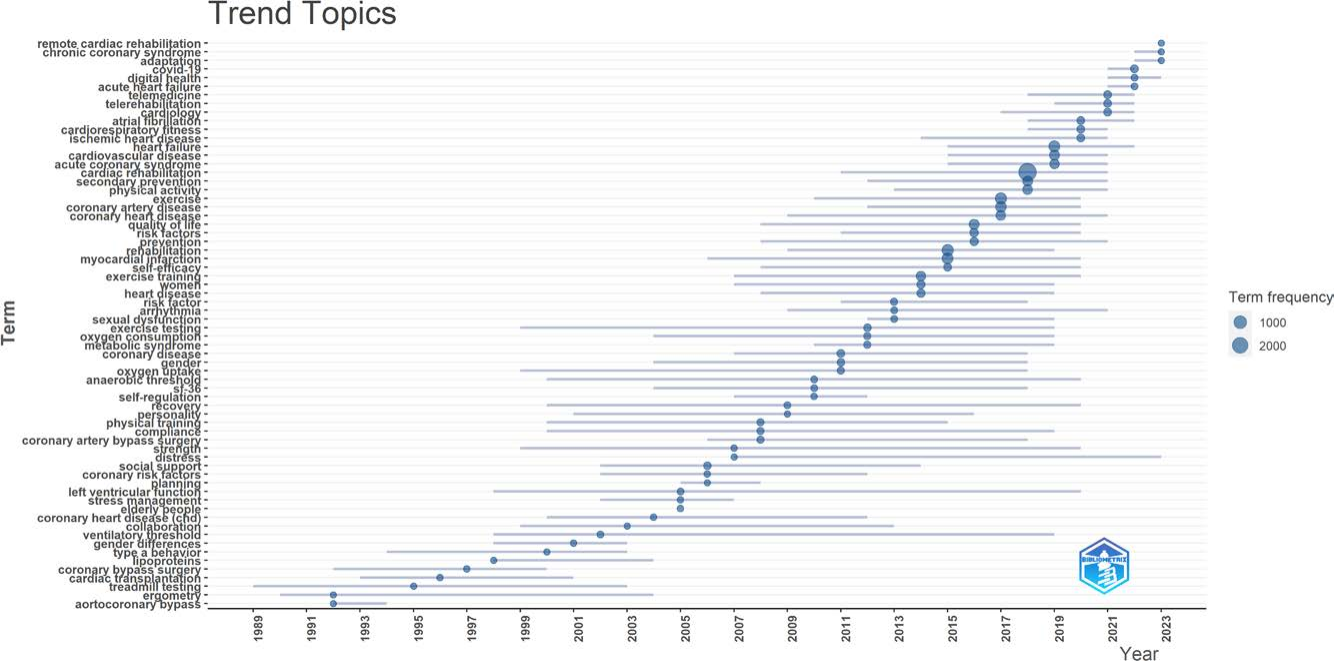
Emerging CRR trend topics from 1989 to 2023. This bubble plot highlights the temporal emergence and frequency of keywords in cardiac rehabilitation research. Recent trends include “telemedicine,” “remote cardiac rehabilitation,” and “COVID-19,” reflecting post-pandemic shifts. Larger bubbles indicate higher term frequencies, showcasing the rise of key themes such as “exercise,” “quality of life,” “depression,” and “heart failure. COVID-19 = coronavirus disease 2019, CRR = cardiac rehabilitation research.

Figure 11 presents the emerging themes of CRR. The identified themes included remote cardiac rehabilitation, adaptation, chronic coronary syndrome, digital health, and COVID-19. These emerging themes reflect the evolving landscape of CRR, emphasizing the importance of technology, customization, and addressing current challenges in healthcare delivery. Further research in these areas could enhance cardiac rehabilitation practice and outcomes.

### 9.3. CRR in the post-COVID-19 era and thematic map

Data for post-COVID-19 data were collected in January 2020. The data for this period represent 30.78% (2824/9173). No changes were observed in the prolific components of CRR. From 2020 to 2023, the occurrences and percentages of keywords in CRR are as follows: “cardiac rehabilitation” appears 1081 times (8.77%), “heart failure” appears 262 times (2.13%), “exercise” appears 244 times (1.98%), “myocardial infarction” appears 198 times (1.61%), “cardiovascular disease” appears 214 times (1.74%), “coronary artery disease” appears 148 times (1.20%), “rehabilitation” appears 131 times (1.06%), “physical activity” appears 126 times (1.02%), and “secondary prevention” appears 123 times (1.00%). These keywords highlighted the prominent areas of focus in CRR during this period, including various aspects of rehabilitation, specific cardiovascular conditions, exercise, mental health, and the impact of COVID-19.

**Figure 12.**
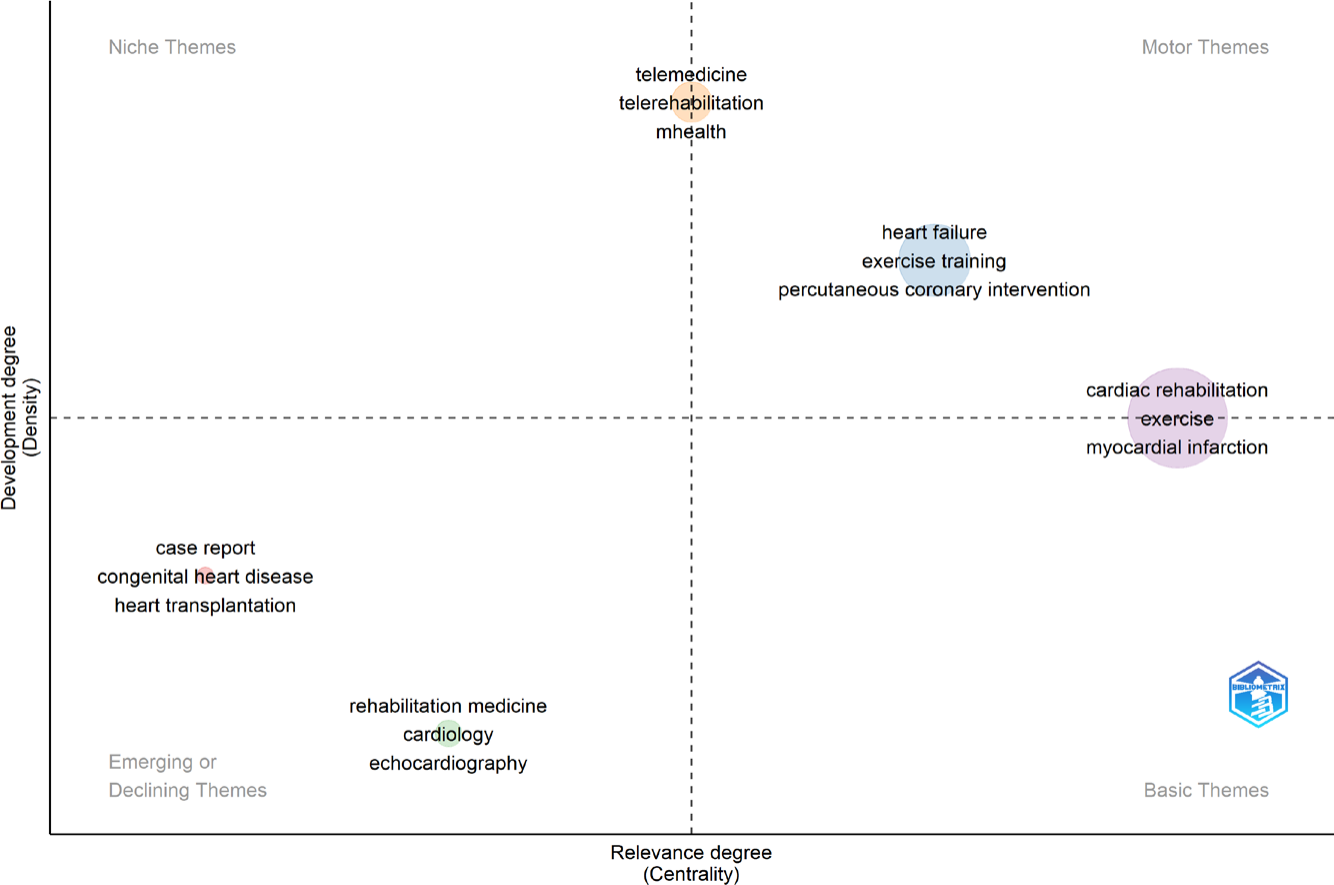
Thematic map of CRR in post-COVID-19 era. COVID-19 = coronavirus disease 2019, CRR = cardiac rehabilitation research.

Figure 12 and Table 4 present a thematic map of the CRR in the post-COVID-19 era from 2020 to 2023. In the post-COVID era, there are 3 prominent news themes that have emerged: “heart failure,” “rehabilitation medicine,” and “telemedicine.” “Rehabilitation” theme has disappeared in the post-COVID-19 era. The theme “cardiac rehabilitation” has moved from the center of the map and now holds higher centrality in the post-COVID era, it indicates an increased importance and relevance of cardiac rehabilitation in research, practice, and potentially in news coverage.

**Table 4:** Clusters, centrality, density, and keywords, and classification of CRR in post-COVID-19 era.

Clusters	Callon centrality	Callon density	Keywords of the theme	Classification
Case report	0.02	4.92	Case report, congenital heart disease, heart transplantation	Emerging
Heart failure	0.23	5.72	Heart failure, exercise training, percutaneous coronary intervention, exercise capacity, cardiorespiratory fitness, mortality, atrial fibrillation, frailty, functional capacity, chronic heart failure, high-intensity interval training, prognosis, elderly, physical function, cardiopulmonary exercise test, aerobic exercise, exercise test, resistance training, aging, blood pressure, cardiopulmonary exercise testing, heart rate, acute heart failure, epidemiology, activities of daily living, exercise prescription, exercise therapy, peak oxygen uptake, survival	Motor
Rehabilitation medicine	0.05	4.56	Rehabilitation medicine, cardiology, echocardiography, ischemic heart disease	Emerging
Cardiac rehabilitation	0.48	5.37	cardiac rehabilitation, exercise, myocardial infarction, cardiovascular disease, coronary artery disease, rehabilitation, physical activity, secondary prevention, coronary heart disease, acute coronary syndrome, quality of life, depression, COVID-19, cardiovascular diseases, anxiety, adherence, acute myocardial infarction, risk factors, ischemic heart disease, cardiac surgery, hypertension, qualitative research, obesity, prevention, cardiovascular rehabilitation, health-related quality of life, stroke, exercise tolerance, mental health, randomized controlled trial, self-efficacy, diabetes, heart disease, meta-analysis, barriers, lifestyle, women, diabetes mellitus, nursing, self-management, systematic review, older adults, coronary disease, education, outcomes, pulmonary rehabilitation, smoking, cardiovascular risk factors, coronary artery bypass grafting, feasibility, patient education, physical therapy, return to work, self-care	Delineating between motor and basic themes
Telemedicine	0.09	6.25	Telemedicine, telerehabilitation, mhealth, telehealth, digital health, mobile health, cardiovascular, home-based, physical exercise	Delineating between motor and niche themes

COVID-19 = coronavirus disease 2019, CRR = cardiac rehabilitation research.

### 9.4. Dynamicity of CRR in post-COVID-19 era

**Figure 13.**
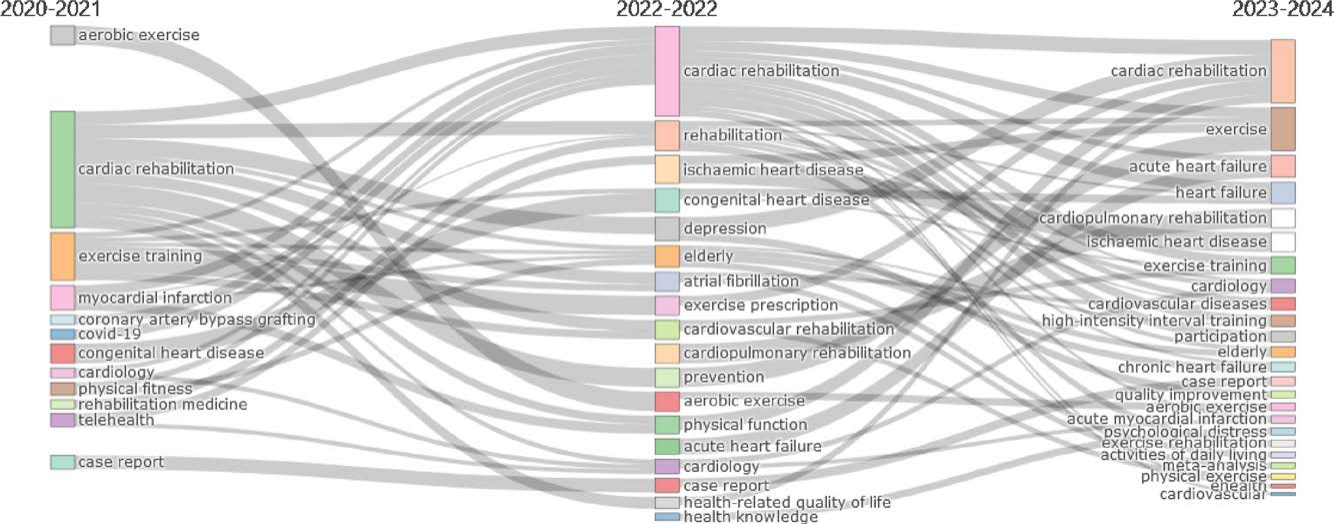
Dynamicity of CRR in post-COVID-19 era. COVID-19 = coronavirus disease 2019, CRR = cardiac rehabilitation research.

Figure 13 illustrates the thematic evolution of CRR over time. The analysis revealed shifts in research focus and emerging themes within specific time periods. From 2020 to 2021, the predominant themes in CRR were aerobic exercise, cardiac rehabilitation, cardiology, depression, elderly patients, health-related quality of life, physical function, prevention, rehabilitation, and specific cardiac conditions such as atrial fibrillation and myocardial infarction. In 2022, research on cardiac rehabilitation, cardiology, and specific conditions such as congenital heart disease and coronary artery bypass grafting continued. Additionally, new themes emerged, including cardiovascular rehabilitation, exercise training, telehealth, and topics related to COVID-19. Looking ahead to 2023 to 2024, the thematic evolution suggests a further expansion of areas such as aerobic exercise, atrial fibrillation, acute heart failure, acute myocardial infarction, cardiovascular diseases, electronic health (e-health), exercise, heart failure, high-intensity interval training, meta-analysis, participation, psychological distress, quality improvement, and specific patient populations, such as the elderly and those with chronic heart failure. These evolving themes indicate the dynamic nature of CRR research, focusing on exploring new interventions, optimizing rehabilitation strategies, addressing specific cardiac conditions, and considering psychological and quality-of-life aspects. This analysis provides valuable insights for researchers and practitioners to remain informed about emerging trends.

### 9.5. Emerging themes in post-COVID-19 era

**Figure 14.**
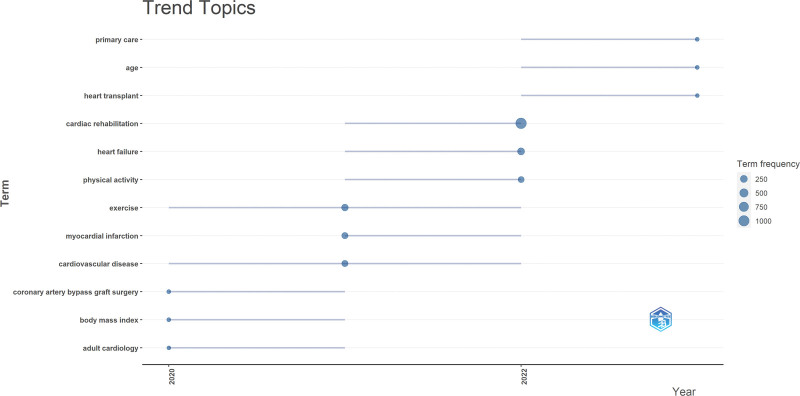
Emerging themes in post-COVID-19 era. COVID-19 = coronavirus disease 2019.

The emerging themes in Figure 14 demonstrate the shifting priorities and focus areas in the post-COVID-19 era. The emphasis on age highlights the need for tailored healthcare approaches based on different age groups, whereas primary care underscores the importance of comprehensive and accessible healthcare services. The heart transplant theme suggests ongoing advancements and research in cardiac transplantation, aiming to improve patient outcomes and address the complexities of this procedure.

## 10. Discussion

This study aimed to provide a comprehensive bibliometric analysis of CRR, highlighting scientific progress, spatial distribution, keyword trends, thematic evolution, and research gaps. It includes a focused quantitative and thematic assessment of literature published during the post-COVID-19 era (2020–2023) to explore emerging priorities and shifts in CRR research. This study sought to provide insights into the impact of COVID-19 on CRR practices, identify areas of research and clinical focus, and contribute to the understanding of the current state and future directions of the pandemic. The increase in the number of publications on CRR in recent years can be attributed to several factors. First, there is growing awareness of the importance of cardiac rehabilitation in improving patient outcomes and quality of life.^[20]^ Advancements in treatment and technology have expanded the possibilities and effectiveness of cardiac rehabilitation, driving researchers to explore their impact.^[20]^ Increased research funding, collaboration between researchers and institutions, and the influence of policies and guidelines have also contributed to the rise in publications.^[21]^ The emphasis on evidence-based practice in healthcare, positive patient outcomes, cost-effectiveness of cardiac rehabilitation, and the global aging population’s increased demand for effective programs further motivates researchers to study and publish in this field.^[22]^ Overall, these factors reflect a concerted effort to enhance the understanding, delivery, and outcomes of cardiac rehabilitation interventions.

It has been reported that the author Grace, S.L. has made notable contributions to various topics between 2018 and 2022. Her research focuses on heart rehabilitation, cardiovascular disease, and the concept of metabolic equivalents. Additionally, she explored the realm of patient care, particularly in relation to medical students, and the utilization of bismuth subnitrate. Another significant area of their research was heart rehabilitation, with an emphasis on understanding the impact of anxiety in individuals with ischemic heart disease. These contributions highlight Grace’s dedication to advancing knowledge and improving patient care in the field of heart rehabilitation, addressing both the medical and psychological aspects of cardiovascular health.^[23–28]^

The dominance of institutions, such as the University of Toronto and its affiliated entities, including the University Health Network and Toronto Rehabilitation Institute, reflects Canada’s central role in advancing CRR. These institutions have not only led in terms of publication volume, but have also fostered a strong ecosystem of clinical and academic collaboration, particularly in the rehabilitation sciences. Similarly, York University and the Mayo Clinic are recognized for their consistent contributions, further emphasizing the role of research-intensive institutions in shaping the CRR landscape.

At the country level, the United States has emerged as the most productive and influential contributor, leading to both publication volume and citation impact. This underscores the country’s sustained investment in cardiovascular research supported by strong funding structures, multi-institutional collaborations, and advanced healthcare infrastructure, Canada and the United Kingdom also demonstrated a significant impact, both in publication numbers and collaborative ties, reflecting their commitment to evidence-based rehabilitation practices. The inclusion of Australia, Japan, and European nations such as Italy and Germany among the top contributors highlights the global reach and growing interest in the CRR. High citation counts from these countries reflect the quality and influence of their research. Notably, Brazil’s high MCP ratio signals its strategic engagement in international collaborations, which is vital for capacity building and cross-context knowledge transfer, especially in emerging economies. Collaboration network mapping also revealed that North America and Western Europe remain the primary hubs for CRR research, while Asia-Pacific countries are becoming increasingly active contributors.^[29,30]^ The strong bilateral collaborations between the United State–Canada, United State–United Kingdom, and United State–Italy point to shared research priorities and well-established academic exchanges. These findings highlight the importance of institutional excellence and international collaboration in driving the evolution of CRR. They also indicate potential opportunities for expanding global partnerships and fostering CRR capacity in underrepresented regions, ultimately contributing to more inclusive and diverse research outcomes in cardiovascular health.

The application of Bradford’s and Lotka’s Laws in CRR provides significant insights with practical implications. Bradford’s Law shows that a small core of 30 journals accounts for a large proportion of CRR publications, suggesting that researchers can enhance visibility and impact by targeting these sources, while librarians can prioritize them for subscriptions. Lotka’s Law highlights that a small number of prolific authors produce most of the literature, with 74.6% of authors contributing only one paper. This underscores the importance of supporting leading researchers and fostering collaboration networks while encouraging broader participation from the academic community. Together, these findings can inform future research strategies, funding priorities, institutional planning, and policymaking in the CRR domain.

Themes in a thematic map can shift because of factors such as evolving research focus, emerging trends and priorities, changes in public interest, and external factors and context. These shifts reflect changes in the prominence and relevance of various topics in a field. The 4 components of the thematic map are themes (key issues or subject areas), relationships (connections between themes), centrality (importance or prominence of themes), and spatial representation (visual display of themes and their relationships). Thematic maps provide insights into the patterns and dynamics within a field, helping researchers and viewers to understand the evolving landscape of a subject area.^[31,32]^ The theme “cardiac rehabilitation” has moved from the center of the map and now holds higher centrality in the post-COVID era; it indicates an increased importance and relevance of cardiac rehabilitation in research, practice, and potentially in news coverage.^[1,33]^ This shift suggests a greater recognition of the value of cardiac rehabilitation in promoting recovery, improving outcomes, and enhancing the quality of life of individuals with cardiovascular conditions in the post-COVID era.^[33,34]^ The higher centrality of this theme signifies a growing focus on understanding and implementing effective cardiac rehabilitation programs, integrating rehabilitation into healthcare systems, and advancing the field through research and innovation. The increased relevance of cardiac rehabilitation highlights its vital role in addressing the long-term effects of cardiovascular diseases and supporting individuals in their recovery journey.^[35]^ This emphasizes the importance of multidisciplinary care, exercise training, lifestyle modifications, and risk factor management in improving cardiovascular health. As cardiac rehabilitation gained higher centrality in the thematic map, it underscored the significance of this therapeutic intervention and its potential impact on the post-COVID era’s cardiovascular healthcare landscape.

In the post-COVID-19 era, a new theme has emerged in the thematic map, encompassing “telemedicine,” “telerehabilitation,” “mobile health,” “telehealth,” “digital health,” “mobile health,” “cardiovascular health,” “home-based care,” and “physical exercise.” This theme reflects the growing importance of technology-driven approaches to health care delivery and cardiovascular management. Telemedicine, telerehabilitation, and telehealth utilize telecommunication technology to provide remote healthcare services, including consultation, monitoring, and rehabilitation.^[36]^ Mobile health and mobile health focus on using mobile devices for health monitoring and management, whereas digital health encompasses various digital tools for healthcare delivery.^[36]^ The inclusion of cardiovascular health highlights the relevance of these technologies in managing heart-related conditions. Additionally, the theme emphasizes home-based care and the significance of physical exercise in promoting cardiovascular health, leveraging technology, and telehealth to enable individuals to remotely engage in guided exercise programs remotely.^[37]^ Overall, this emerging theme underscores the potential of technology-enabled approaches to enhance access to healthcare services, provide remote support, and promote physical activity in cardiovascular care post-COVID-19.

The keywords of the theme include “case report,” “congenital heart disease,” and “heart transplantation.” These keywords represent the main topics or subject areas associated with the themes of the case reports in the context of congenital heart disease and heart transplantation. The Callon centrality of 0.02 suggests that the theme of “case report” is not highly prominent or central within the thematic map. This indicates that there may be other themes with a higher centrality and influence in the field. The Callon density of 4.92 signifies a relatively high density of connections within the thematic map. This suggests that there is a strong relationship between the keywords. The classification of the theme as “Emerging” indicates that it is a relatively new or evolving area within the field with potential for further development and exploration.

The changing landscape of CRR in the post-COVID-19 era underscores evolving priorities within the field. From 2020 to 2021, the identified themes highlighted a strong focus on the fundamental aspects of CRR. Topics such as aerobic exercise, cardiac rehabilitation, and cardiology took center stage during this period, indicating a concerted effort to comprehend the impact of exercise and rehabilitation interventions on cardiovascular health. The inclusion of specific conditions such as atrial fibrillation and myocardial infarction suggests a dedicated exploration of tailored rehabilitation approaches for patients with these cardiac ailments. Additionally, research on depression, quality of life, and physical function underscores the recognition of the psychological and functional aspects of cardiac rehabilitation programs.^[37,38]^ In 2022, the thematic landscape will expand further, reflecting emerging areas of interest and addressing evolving challenges. The inclusion of topics such as telehealth and COVID-19 indicates the influence of technological advancements and adaptation of rehabilitation practices during the global pandemic.^[39]^ Exploration of cardiovascular rehabilitation highlights the broader scope of interventions beyond traditional cardiac rehabilitation, potentially encompassing a wider range of cardiovascular conditions.^[40]^ The presence of specific conditions such as congenital heart disease and coronary artery bypass grafting suggests a growing focus on specialized rehabilitation approaches for these patient populations. Looking forward to 2023 to 2024, the thematic evolution suggests continued diversification and exploration of new frontiers in the CRR.^[41]^ Emerging themes such as acute heart failure, acute myocardial infarction, and high-intensity interval training indicate an increasing emphasis on addressing acute cardiovascular events and optimizing rehabilitation strategies to improve outcome.^[42]^ The inclusion of topics like psychological distress, quality improvement, and e-health points to the recognition of holistic patient care, incorporating mental health aspects, advancements in healthcare technology, and pursuit of quality-driven rehabilitation practice.^[43]^ Clinically, the rise of telemedicine as a theme supports the integration of remote, accessible, and scalable rehabilitation services – especially critical during and after pandemic conditions. The prominence of aging as a research theme emphasizes the importance of customizing rehabilitation for older adults who may present unique physical limitations and comorbidities. Similarly, increased attention to heart transplantation research highlights the need for specialized rehabilitation protocols for transplant recipients to enhance recovery, manage complications, and support long-term outcomes. Overall, the thematic evolution of the CRR reflects a progressive and multifaceted research landscape. The identified themes demonstrate a concerted effort to advance knowledge, refine interventions, and address the evolving needs of patients undergoing cardiac rehabilitation. This analysis provides valuable insights for researchers, practitioners, and policymakers to remain informed about the current trends and future directions in the field, ultimately contributing to the development of evidence-based practices and improved patient outcomes in cardiac rehabilitation.

Our thematic shifts align with recent policy and guideline directions: the rise of home-based/hybrid CR parallels expanded telehealth reimbursement in several jurisdictions; the emphasis on primary care interfaces is consistent with WHO initiatives (e.g., Rehabilitation 2030) and national NCD strategies integrating rehabilitation into primary health care; and aging-focused themes echo calls to tailor CR for older adults. Geographic gaps also underscore the need for implementation financing and workforce development in LMICs.

Emerging themes in the post-COVID-19 era are shown in Figure 14. In this period, there is an increasing recognition of the need for tailored healthcare approaches based on different age groups.^[44]^ Age-related considerations in cardiac rehabilitation are crucial, as older adults often have unique physiological needs, higher comorbidity burdens, and greater susceptibility to functional decline during recovery.^[45]^ Understanding how age influences treatment approaches, outcomes, and rehabilitation strategies can help optimize care for different age groups.^[45,46]^ From a clinical standpoint, this necessitates the development of age-specific rehabilitation protocols that address mobility limitations, medication management, and psychological support. Programs focusing on geriatric rehabilitation and frailty mitigation can significantly enhance long-term outcomes in aging populations.

The emphasis on primary care reflects the growing importance of comprehensive and accessible healthcare services in the post-pandemic era. Primary care plays a vital role in managing cardiovascular health, including prevention, early detection, and coordination of cardiac rehabilitation services.^[47,48]^ Clinically, integrating cardiac rehabilitation into primary care settings – through multidisciplinary teams or referral systems – can improve continuity of care and reduce readmissions. It also enables early identification of cardiovascular risk factors, allowing interventions before adverse events occur.

The presence of the heart transplant theme suggests ongoing advancements and research in cardiac transplantation. Heart transplantation is a complex procedure with specific considerations for patient selection, posttransplant care, and long-term outcomes.^[49]^ Clinical practice, this highlights the importance of posttransplant rehabilitation programs that address immunosuppression-related complications, physical reconditioning, and psychosocial adaptation. Developing tailored rehabilitation strategies for transplant patients may improve survival rates and quality of life. Furthermore, emerging themes like telemedicine, digital health, and e-health point to a paradigm shift in cardiac rehabilitation delivery. These technologies allow for home-based and remote monitoring programs, reducing barriers related to travel, time, and clinic access. For practitioners, implementing tele-rehabilitation platforms with real-time feedback and remote supervision can enhance patient adherence and scalability of services, particularly in underserved regions. These shifting priorities and areas of focus reflect the evolving landscape of CRR in the post-COVID-19 era. They underscore the need for personalized approaches, expanded primary care involvement, technological integration, and specialized protocols to optimize cardiac care and ultimately improve clinical outcomes.

## 11. Limitations

This study has several limitations that should be taken into account. Firstly, it relies solely on the Scopus database, which may not include all relevant publications, particularly those indexed in other databases such as PubMed or Web of Science. Secondly, only articles published in English were included, potentially introducing language bias and excluding valuable research published in other languages. Thirdly, due to the timing of data retrieval and the extended correspondence with the journal, the dataset includes records only up to the year 2023. Additionally, the collaboration analysis is limited by the methodology used, and the study does not include a detailed qualitative evaluation of the content and real-world impact of the research. These limitations should be considered when interpreting the study findings. As a bibliometric analysis, our findings reflect publication volume, influence, and thematic patterns rather than methodological quality or clinical effectiveness. Bibliometric indicators (e.g., citations, centrality) are proxies of attention, not evidence of benefit. Accordingly, these trends should inform – but not substitute for – rigorous quality appraisal and outcome-focused research.

## 12. Conclusions

This study provides valuable insights into the research productivity, citation impact, and emerging themes in the field of CRR. The findings highlight the leading countries in terms of research output and collaboration networks, emphasizing the importance of international partnerships in advancing CRR. Key themes identified in the post-COVID-19 era include remote and virtual cardiac rehabilitation, psychological well-being, health disparities, and the long-term effects of COVID-19 on cardiac function. These reflect the shifting priorities in CRR and reveal a notable research gap – specifically, the lack of comprehensive analysis of how these evolving themes are integrated into clinical outcomes, practice guidelines, and real-world implementation strategies. The study also underscores the limited qualitative synthesis in the literature and underrepresentation of low- and middle-income countries in CRR research. While telemedicine, aging, and home-based rehabilitation emerge as priorities, delineating specific research questions and programmatic changes lies beyond this study’s scope. We recommend subsequent consensus-driven agenda setting (e.g., Delphi) to translate these themes into testable questions and implementation strategies. The implications of this study are significant for healthcare professionals, policymakers, and researchers. The findings highlight the need for age-specific and culturally sensitive interventions, strengthened primary care infrastructure, innovation in cardiac transplant protocols, and greater emphasis on mental health within CRR programs. By identifying core journals, prolific authors, and collaborative networks, this study also provides practical direction for researchers seeking to publish and collaborate effectively in this domain. Future research should focus on integrating bibliometric trends with clinical data to assess the translational value of scientific outputs, expand the analysis to include underrepresented regions, and adopt mixed methods approaches that combine bibliometric mapping with qualitative content analysis. Additionally, developing global frameworks for standardized reporting and best practices in virtual CRR can enhance the delivery and evaluation of cardiac rehabilitation services worldwide.

## Acknowledgments

We would like to thank Princess Nourah bint Abdulrahman University for supporting this project through Princess Nourah bint Abdulrahman University Researchers Supporting Project number (PNURSP2025R146), Princess Nourah bint Abdulrahman University, Riyadh, Saudi Arabia.

## Author contributions

**Conceptualization:** Siddig Ibrahim Abdelwahab, Manal Mohamed Elhassan Taha, Hadeel R. Bakhsh, Monira I. Aldhahi.

**Data curation:** Siddig Ibrahim Abdelwahab, Manal Mohamed Elhassan Taha.

**Formal analysis:** Siddig Ibrahim Abdelwahab, Manal Mohamed Elhassan Taha, Monira I. Aldhahi.

**Funding acquisition:** Siddig Ibrahim Abdelwahab, Manal Mohamed Elhassan Taha, Monira I. Aldhahi.

**Investigation:** Siddig Ibrahim Abdelwahab, Manal Mohamed Elhassan Taha, Monira I. Aldhahi.

**Methodology:** Siddig Ibrahim Abdelwahab, Manal Mohamed Elhassan Taha, Monira I. Aldhahi.

**Project administration:** Siddig Ibrahim Abdelwahab, Manal Mohamed Elhassan Taha, Monira I. Aldhahi.

**Resources:** Siddig Ibrahim Abdelwahab, Manal Mohamed Elhassan Taha, Hadeel R. Bakhsh, Monira I. Aldhahi.

**Software:** Siddig Ibrahim Abdelwahab, Manal Mohamed Elhassan Taha, Monira I. Aldhahi.

**Supervision:** Siddig Ibrahim Abdelwahab, Manal Mohamed Elhassan Taha.

**Validation:** Siddig Ibrahim Abdelwahab, Manal Mohamed Elhassan Taha, Monira I. Aldhahi.

**Visualization:** Siddig Ibrahim Abdelwahab, Manal Mohamed Elhassan Taha, Hadeel R. Bakhsh, Monira I. Aldhahi.

**Writing – original draft:** Siddig Ibrahim Abdelwahab, Manal Mohamed Elhassan Taha, Hadeel R. Bakhsh, Monira I. Aldhahi.

**Writing – review & editing:** Siddig Ibrahim Abdelwahab, Manal Mohamed Elhassan Taha, Hadeel R. Bakhsh, Monira I. Aldhahi.
